# Methodological Considerations on COVID-19 Mortality in Cancer Patients: A Systematic Review and Meta-Analysis

**DOI:** 10.1093/jncics/pkac063

**Published:** 2022-09-01

**Authors:** Makda Getachew Zewde, Naomi Alpert, Emanuela Taioli

**Affiliations:** Institute for Translational Epidemiology, Icahn School of Medicine at Mount Sinai, New York, NY, USA; Institute for Translational Epidemiology, Icahn School of Medicine at Mount Sinai, New York, NY, USA; Institute for Translational Epidemiology, Icahn School of Medicine at Mount Sinai, New York, NY, USA; Tisch Cancer Institute, Icahn School of Medicine at Mount Sinai, New York, NY, USA; Department of Thoracic Surgery, Icahn School of Medicine at Mount Sinai, New York, NY, USA

## Abstract

**Background:**

Patients with cancer are at risk for severe COVID-19. Previous studies examining mortality in cancer patients with COVID-19 have produced inconclusive results. Several published meta-analyses have aimed to estimate this association; however, because of methodological limitations in study selection and data aggregation, these studies do not reliably estimate the independent association between cancer and COVID-19 mortality. We conducted this systematic review and meta-analysis to determine whether cancer is an independent risk factor for COVID-19 mortality.

**Methods:**

A literature search was performed in PubMed to identify studies that compared COVID-19 mortality in adult patients with and without cancer. Selection criteria included polymerase chain reaction–confirmed COVID-19, multivariate adjustment and/or matching for mortality risk estimates, and inclusion of hospitalized noncancer controls. Adjusted odds ratios and/or hazard ratios for mortality based on cancer status were extracted. Odds ratio and hazard ratio estimates were pooled using a random effects model.

**Results:**

The analysis included 42 studies comprising 129 840 patients: 8612 cancer patients and 121 228 noncancer patients. Of these studies, 18 showed a null difference in survival between cancer and noncancer patients with COVID-19, and 24 studies showed statistically significantly worse survival in cancer patients with COVID-19. Meta-analysis revealed an increased risk of mortality in patients with cancer compared with noncancer patients with COVID-19 (odds ratio = 1.93, 95% confidence interval = 1.55 to 2.41; hazard ratio = 1.54, 95% confidence interval = 1.29 to 1.84).

**Conclusion:**

We conclude that cancer is an independent risk factor for mortality in unvaccinated patients admitted for or diagnosed with COVID-19 during hospitalization.

Since the first reported case of COVID-19 in December 2019, more than 460 million confirmed cases and more than 6 million deaths have been attributed to the infection (1). The clinical presentation of COVID-19 is highly variable and ranges from asymptomatic to mild to severe. Early studies have aimed to identify high-risk patient populations with the goal of tailoring clinical treatment for patients at risk of severe disease and mortality. Several comorbidities, including hypertension, diabetes, chronic obstructive pulmonary disease, and congestive heart failure, have been found to be associated with severe disease in COVID-19 patients (2-5).

Patients with cancer are a heterogenous population hypothesized to have multiple risk factors for severe disease and complications, owing to their hypercoagulable state, altered immune response, the immunosuppressive effects of chemotherapy, and/or the immunosuppressive regimens required for patients with hematologic malignancies undergoing hematopoietic cell transplantation (6-8). Numerous studies have examined the risk of mortality in cancer patients with COVID-19; however, many of these studies were limited by single institution data and small sample size and results thus far have been inconclusive (9-11). We have previously discussed in detail the methodological limitations of published data (12,13) and have noted that published studies are limited by a lack of patient controls or the inclusion of inappropriate controls such as COVID-19 positive outpatients, hospitalized health-care workers, or patients with other types of cancer and that many of these biases in study design can generate inflated risk estimates.

Several meta-analyses assessing mortality in COVID-19 patients with cancer have been published in an attempt to obtain more precise mortality risk estimates and have shown a statistically significantly increased risk of death among cancer patients with COVID-19. However, these meta-analyses demonstrate methodological flaws in study selection and data aggregation. In an earlier meta-analysis by Zhang et al. (13), only 3 of 15 studies included noncancer controls, and only 2 among these 3 studies reported adjusted survival estimates. In Tian colleagues’ meta-analysis (14), only 1 of 8 included studies reported adjusted estimates. Yang et al. (15) published a meta-analysis of 10 studies; however, only 3 reported adjusted or matched estimates, and in 1 study with both crude and adjusted estimates, only the crude estimate was used for the meta-analysis. Additionally, studies with both clinical and laboratory COVID-19 confirmation were included (15). In Venkatesulu and colleagues’ meta-analysis (16), only 3 of 10 studies reported adjusted estimates, and among these, 1 study included mortality and severe events in the outcome variable. Requirement for polymerase chain reaction (PCR)–confirmed COVID-19 infection was not an inclusion criteria, and 3 of the 10 studies either did not describe their control group or used inappropriate controls (16). Di Felice et al. (17) published a meta-analysis of 27 studies in which only 14 of the 27 studies reported adjusted estimates, and laboratory-confirmed infection was not among the inclusion criteria. Most recently, Han et al. (18) published a meta-analysis of 7 studies, all of which used the general population as a control group rather than hospitalized patients without cancer, and only unadjusted estimates were used in the meta-analysis.

Given the methodological limitations in published studies and meta-analyses as described above, it remains unclear whether cancer is an independent risk factor for mortality in COVID-19 patients, and a quantitative estimate of such risk is still uncertain. We conducted this systematic review and meta-analysis to study the impact of cancer status on mortality in patients with laboratory-confirmed COVID-19 prior to the availability of COVID-19 vaccination to the public.

## Methods

### Search Strategy and Inclusion Criteria

**Figure 1.**
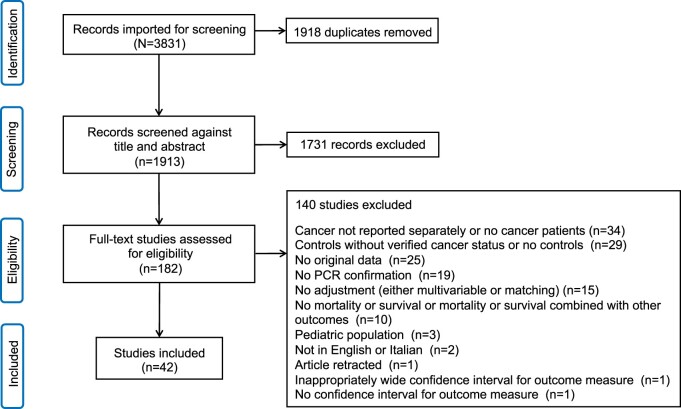
PRISMA diagram of articles included in the meta-analysis. PCR = polymerase chain reaction.

A literature search was performed in PubMed using the search terms (“Sars-cov-2” OR “Covid” OR “COVID-19”) AND (“Cancer” OR “malignancy”) AND (“Mortality” OR “Survival”), and (“Sars-cov-2” OR “Covid” OR “COVID-19”) AND (“Cancer” OR “malignancy” OR “neoplasm”) AND (“outcome” OR “Mortality” OR “Survival”) through February 2021, when vaccination became available to cancer patients. Inclusion criteria were as follows: 1) hospitalized controls were included, 2) COVID cases were PCR confirmed, 3) multivariate or other study adjustment was used, and 4) mortality and survival was reported as an outcome. Exclusion criteria were as follows: 1) reported mortality was combined with other outcomes, 2) absence of controls, or 3) controls came from the general population. The study selection according to PRISMA is reported in Figure 1.

The search returned 3831 publications of which 1918 were duplicates. After an abstract review, 1731 studies were excluded because they were not pertinent, leaving 182 articles to review as full text. Of these, 140 studies were excluded because cancer was not reported separately or no cancer patients were included (n = 34), controls did not have verified cancer status/there were no controls (n = 29), the study was a review or commentary and did not contain original data (n = 25), no PCR confirmation was used (n = 19), no adjustment (either multivariable or matching) was used (n = 15), no mortality/survival was reported or mortality/survival was combined with other outcomes (n = 10), the study included pediatric patients (n = 3), the study was not published in English or Italian (n = 2), the article was retracted (n = 1), confidence intervals for outcome measures were inappropriately wide (n = 1), or confidence intervals were not reported for the outcome (n = 1). The remaining 42 studies were included in this analysis.

### Data Extraction

Descriptive information was extracted from each included study, including author, year of publication, country of publication, study design, study duration, number of cancer and control patients, type of cancer, and variables used for adjustment or matching. Quantitative data extracted from each study included odds ratios (ORs) and/or hazard ratios (HRs) for mortality based on cancer status. If an odds ratio or hazard ratio was not reported, we calculated an odds ratio based on available information in studies that matched their cancer and noncancer cohorts on demographic or clinical variables. Data were extracted independently by 2 reviewers (MZ, NA). In cases of disagreement during data extraction, a final decision was made by a third reviewer (ET). The 2018 Mixed Methods Appraisal Tool was used by 2 researchers (MZ, NA) to perform a quality assessment (Supplementary Methods, available online) (19).

### Statistical Analysis

We summarized the odds ratios or hazard ratios and their 95% confidence intervals (CIs) to assess the association between mortality and cancer status in patients with COVID-19. A random effects model was used to pool odds ratio or hazard ratio estimates. Sensitivity analyses were performed based on study location (Asia, Europe, United States), adjustment methods (multivariable adjustment vs matching), among cohort studies, and among patients with hematologic malignancies. *Q* and *I*^2^ statistics were used to test heterogeneity across studies (20,21). For all tests, 2-sided *P* values less than .05 were considered statistically significant. All analyses were performed using R statistical package, version 3.6.2. (R Core Team 2019).

## Results

### Summary of Included Studies

Table 1 summarizes the design characteristics of each study: 35 studies were cohort studies, 5 were case series, and 2 were cross-sectional studies. The Mixed Methods Appraisal Tool scores of the included studies ranged from 3 to 5 out of a possible 5, and the overall mean score was 4.33 with a standard deviation of 0.46 (Supplementary Table 1, available online). Ten studies were conducted in Asia, 33 in Europe, and 11 in the United States. Turkey was considered both an Asian and European country for sensitivity analysis. The majority of studies (n = 35) included mixed or unspecified cancers in their cancer cohort, and 7 studies included only patients with hematologic malignancies.

**Table 1.** Studies included in the meta-analysis^a^

Study	Study design	Country	No. of patients	No. of cancer patients	No. of noncancer patients	Type of cancer	Variables used for adjustment and/or matching
Martínez-Lopez et al. (49)	Case series	Spain	334	167	167	Hematologic malignancy	Age, sex
Mehta et al. (10)	Case series	United States	1308	218	1090	Mixed or unspecified	Age, sex
Mirani et al. (56)	Case series	Italy	385	62	323	Mixed or unspecified	Age, sex
Mohamed et al. (29)	Case series	United States	7624	484	7140	Mixed or unspecified	Age, sex, chronic kidney disease, race and ethnicity, smoking, COPD, hypertension, diabetes
Stroppa et al. (32)	Case series	Italy	56	25	31	Mixed or unspecified	Age, sex, pneumonia, antiviral treatment
Alpert et al. (11)	Cohort	United States	5556	421	5135	Mixed or unspecified	Age, sex, comorbidities
Altuntas et al. (39)	Cohort	Turkey	994	497	497	Hematological malignancy	Age, comorbidities
An et al. (34)	Cohort	South Korea	10 237	76	10 161	Mixed or unspecified	Age, sex, income, residence, household type, disability, symptoms, infection route, other underlying comorbidities
Anantharaman et al. (23)	Cohort	United States	4627	33	4380	Mixed or unspecified	Age, sex, race and ethnicity, BMI, Charlson comorbidity index, hypertension, diabetes, smoking, neighborhood deprivation index
Başcı et al. (24)	Cohort	Turkey	64	16	48	Chronic myeloid leukemia	Age, sex, comorbidities
Bellan et al. (40)	Cohort	Italy	407	33	277	Mixed or unspecified	Age, obesity, smoking
Brar et al. (33)	Cohort	United States	585	117	468	Mixed or unspecified	Age, sex, ethnicity, smoking, obesity, diabetes, hypertension, COPD, coronary artery disease, heart failure
Ciceri et al. (54)	Cohort	Italy	410	22	383	Mixed or unspecified	Age, coronary artery disease, radiographic assessment of lung edema score, lymphocyte count
Cui et al. (25)	Cohort	China	836	37	799	Mixed or unspecified	Age, sex, COPD, dyspnea, dizziness, respiratory rate, heart rate, neutrophil, lymphocyte, platelet count, D-dimer, lactate dehydrogenase, albumin, EGFR, hypersensitive troponin I, N-terminal pro-brain natriuretic peptide, C-reactive protein, procalcitonin
Docherty et al. (55)	Cohort	UK	20 133	1743	15 611	Mixed or unspecified	Age, sex, chronic cardiac, pulmonary kidney diseases, diabetes, obesity, chronic neurological disorder, dementia, moderate or severe liver disease
Eshrati et al. (35)	Cohort	Iran	3188	41	3147	Mixed or unspecified	Age, sex, diabetes, liver, cardiovascular, kidney disease, chronic pulmonary disease, chronic nervous disease
Esme et al. (41)	Cohort	Turkey	13 770	1422	12 348	Mixed or unspecified	Age, sex, hypertension, diabetes, heart failure, chronic kidney disease, dementia
Gude-Sampedro et al. (42)	Cohort	Spain	10 454	34	10 420	Hematologic cancers	Age, sex, ischemic heart disease, dementia, COPD, diabetes, chronic kidney disease
Guerra Veloz et al. (26)	Cohort	Spain	226	24	202	Mixed or unspecified	Age, sex, hypertension, congestive heart failure, coronary artery disease, COPD, diabetes, obesity, chronic liver disease
Gupta et al. (43)	Cohort	United States	2215	112	2103	Mixed or unspecified	Age; sex; race; hypertension; diabetes; BMI; coronary artery disease; congestive heart failure; COPD; smoking; <3 days from symptom onset to ICU day 1; PaO2: FiO2, shock on ICU day 1; coagulation; liver; renal component of SOFA score; number of ICU beds
Haase et al. (36)	Cohort	Denmark	323	15	308	Mixed or unspecified	Age, sex, heart failure, hypertension, chronic pulmonary disease, chronic kidney disease, immunocompromised
Huang et al. (37)	Cohort	China	676	33	643	Mixed or unspecified	Age, sex, hypertension, diabetes, heart disease, D-dimer ≥0.5 mg/L, CRP ≥ 10 mg/L, PCT ≥0.5 ng/mL, LDH ≥250 U/L
Iftimie et al. (44)	Cohort	Spain	188	26	162	Mixed or unspecified	Type 2 diabetes, cardiovascular diseases; chronic liver, lung, kidney diseases; chronic neurological diseases; age; sex; smoking; alcohol
Jimenez et al. (45)	Cohort	Spain	1540	103	1437	Mixed or unspecified	Age, sex, neurological disease, chronic kidney disease
Joharatnam-Hogan et al. (27)	Cohort	UK	120	30	90	Mixed or unspecified	Age, sex, number of comorbidities
Kim et al. (46)	Cohort	Republic of Korea	2254	85	2169	Mixed or unspecified	Age, fever, need for O_2_ at admission, diabetes, dementia, heart failure, hypertension, neurological disease, infiltration on chest X-ray at initial diagnosis, BMI > 25, chronic liver disease
Krause et al. (47)	Cohort	United States	85	11	74	Mixed or unspecified	Age, ethnicity, insurance status, BMI, hypertension, diabetes
Kvåle et al. (28)	Cohort	Norway	8809	372	8437	Mixed or unspecified	Age, sex, cardiovascular disease, cancer stage, asthma, dementia, diabetes, obesity, COPD, chronic kidney disease
Lunski et al. (48)	Cohort	United States	5145	312	4833	Mixed or unspecified	Age, sex, race, chronic kidney disease, COPD, coronary artery disease, diabetes, hypertension, obesity
Meng et al. (50)	Cohort	China	2665	109	2556	Mixed or unspecified	Age, sex, hypertension, coronary heart disease, diabetes, COPD, chronic kidney disease, cerebrovascular disease, hepatitis, tuberculosis
Nogueira et al. (30)	Cohort	Portugal	20 293	611	19 682	Mixed or unspecified	Asthma; cardiac disease; chronic hematological disorder; diabetes; HIV or other immune deficiency; kidney, liver, lung, neuromuscular disorder
Poterucha et al. (57)	Cohort	United States	887	37	850	Mixed or unspecified	Age, sex, hypertension, diabetes, chronic kidney disease, primary lung disease, coronary artery disease, obesity, HFrEF, HFpEF, history of cancer, abnormal high-sensitivity cardiac troponin T ≥ 20 ng/L, ECG AF or AFL, abnormal QRS morphology, ST and T wave abnormalities
Raines et al. (31)	Cohort	United States	440	80	360	Mixed or unspecified	Age; sex; race; BMI; immunodeficiency syndromes; pulmonary, GI, renal, hematologic, endocrine, cardiovascular diseases; neurologic problems; smoking
Ramachandran et al. (58)	Cohort	United States	188	53	135	Mixed or unspecified	Aged older than 60 years, hypertension, smoking, hemoglobin, lactate, C-reactive protein, alkaline phosphatase
Sanchez-Pina et al. (51)	Cohort	Spain	92	39	53	Hematologic malignancy	Age, need for oxygen
Shah et al. (59)	Cohort	UK	1183	68	1115	Hematologic malignancy	Age, sex
Shoumariyeh et al. (38)	Cohort	Germany	78	39	39	Mixed or unspecified	Age, CRP, IL-6, renal impairment or CKD, presence of ≥2 organ comorbidities
Sng et al. (60)	Cohort	UK	320	94	226	Mixed or unspecified	Age; sex; south Asian origin; cardiovascular, chronic kidney disease, hypertension, cerebrovascular disease
Thompson et al. (52)	Cohort	UK	470	87	383	Mixed or unspecified	Age, hypertension, admission CRP ≥100 μg/ml, admission platelet count <150 x 10^3^/μl, admission chest radiograph >50% total lung field infiltrates, acute kidney injury
Yigenoglu et al. (53)	Cohort	Turkey	1480	740	740	Hematologic malignancy	Age, sex, comorbidity
Alamdari et al. (22)	Cross-sectional	Iran	459	52	407	Mixed or unspecified	Age, malignancy, magnesium, creatinine, lymphocyte count, c-reactive protein
Zandkarimi et al. (61)	Cross-sectional	Iran	1831	32	1799	Mixed or unspecified	Age, sex, diabetes, weak immune system, coronary heart disease, chronic lung disease, kidney disease

a AF/AFL = atrial fibrillation/atrial flutter; BMI = body mass index; COPD = chronic obstructive pulmonary disease; CRP = c-reactive protein; ECG = electrocardiogram; EGFR = estimated glomerular filtration rate; GI = gastrointestinal; HFpEF = heart failure with preserved ejection fraction; HFrEF = heart failure with reduced ejection fraction; ICU = intensive care unit; IL = interleukin; LDH = lactate dehydrogenase; PCT = procalcitonin; SOFA = sequential organ failure assessment score;.

**Figure 2.**
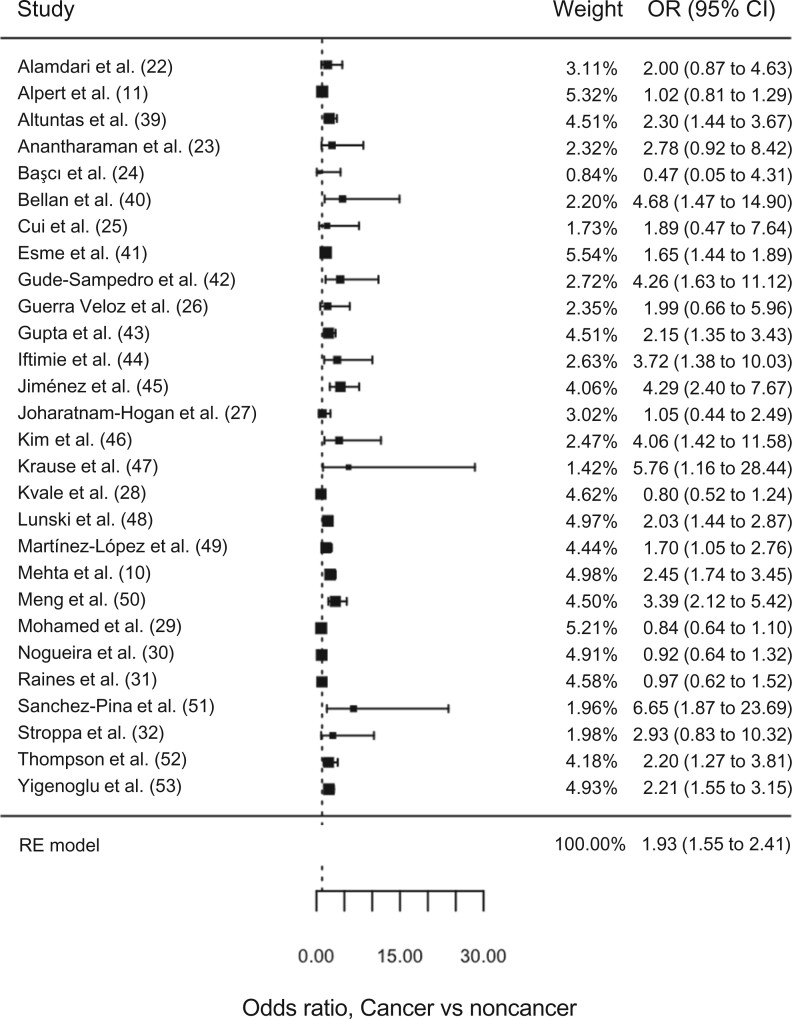
Relative odds of mortality in cancer vs noncancer patients in 28 studies reporting odds ratios for mortality. The reference group is the noncancer group. The 95% confidence intervals are indicated by the error bars. CI = confidence interval; OR = odds ratio; RE = random effects.

**Figure 3.**
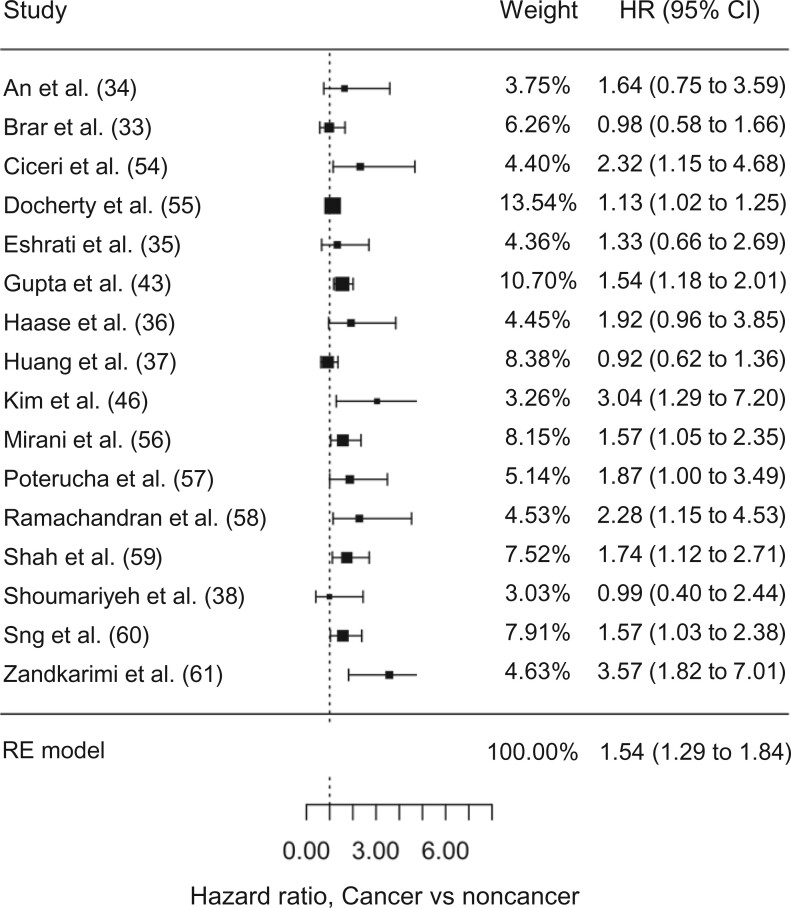
Relative hazard of mortality in cancer vs noncancer patients in 16 studies reporting hazard ratios for mortality. The reference group is the noncancer group. The 95% confidence intervals are indicated by the error bars. CI = confidence interval; HR = hazard ratio; RE = random effects.

The 42 selected studies included 8612 cancer patients and 121 228 noncancer controls (Table 1). Of these, 18 studies showed a null difference in survival between cancer and noncancer patients with COVID-19 (11,22–38), and 24 studies showed statistically significantly worse survival in cancer patients with COVID-19 (10,39–61) (Figures 2 and 3).

### Meta-Analysis

Twenty-six studies reported outcomes in the format of odds ratios of cancer vs noncancer patients, and 14 studies reported hazard ratios. Two studies reported both odds ratios and hazard ratios. Meta-analysis of the included studies (n = 42) was conducted separately for studies reporting either odds ratios (n = 28) or hazard ratios (n = 16). Variables used for adjustment or matching varied greatly across studies (Table 1). Patients with cancer and COVID-19 had increased risk of mortality compared with noncancer patients with COVID-19 (OR_meta_ = 1.93, 95% CI = 1.55 to 2.41; HR_meta_ = 1.54, 95% CI = 1.29 to 1.84, respectively) (Table 2). There was statistically significant heterogeneity between the studies (OR *Q* test, *P* < .001; HR *Q* test = 37.2, *P*  = .001) and evidence of publication bias (OR Egger test, *P*  = .015; HR Egger test, *P* = .01) (Supplementary Figure 2, available online).

**Table 2.** Sensitivity analyses of COVID-19 mortality in cancer vs noncancer patients^a^

Subgroup	No. of studies	Summary OR (95% CI)	Summary HR (95% CI)	*Q* (*df*)	*P*	*I* ^2^, %
Studies reporting odds ratios						
All studies	28	1.93 (1.55 to 2.41)	—	122 (27)	<.001	81.6
Cohort studies	23	2.01 (1.56 to 2.58)	—	92.5 (22)	<.001	81.9
Hematologic malignancies	6	2.23 (1.77 to 2.81)	—	7.72 (5)	.17	0.00
Asia	5	2.17 (1.68 to 2.81)	—	14.6 (7)	.04	48.1
Europe	16	2.02 (1.49 to 2.73)	—	53.5 (15)	<.001	79.4
United States	8	1.58 (1.09 to 2.29)	—	45.0 (7)	<.001	84.7
Studies adjusting with multivariable models	21	1.99 (1.50 to 2.64)	—	104.9 (20)	<.001	82.3
Matched studies	7	2.12 (1.75 to 2.56)	—	6.22 (6)	.399	0.00
Unadjusted estimates	22	2.80 (2.23 to 3.52)	—	114 (21)	<.001	84.3
Studies reporting hazard ratios						
All studies	16	—	1.54 (1.29 to 1.84)	37.2 (15)	.001	58.1
Cohort studies	14	—	1.45 (1.22 to 1.73)	26.7 (13)	.01	50.2
Asia	5	—	1.76 (1.03 to 3.01)	15.1 (4)	.005	70.0
Europe	7	—	1.46 (1.17 to 1.82)	12.8 (6)	.005	48.0
United States	4	—	1.52 (1.18 to 1.97)	4.42 (3)	.02	19.3
Studies adjusting with multivariable models	16	—	1.54 (1.29 to 1.84)	37.2 (15)	.001	58.1
Unadjusted estimates	7	—	1.84 (1.44 to 2.35)	15 (6)	.02	54.4

a The reference group is noncancer patients. “—” indicates that the odds ratio or hazard ratio is not applicable. CI = confidence interval; HR = hazard ratio; *df *= degrees of freedom; OR = odds ratio; *Q* = *Q* statistic.

To evaluate cancer-specific or region-specific differences in outcomes, we conducted sensitivity analyses in patients with hematologic malignancies and based on study location (Asia, Europe, or United States). The survival differential was greater in patients with hematologic malignancies (OR_meta_ = 2.23, 95% CI = 1.77 to 2.81), without evidence of heterogeneity (*Q* test, *P*  = .172) or publication bias (Egger test, *P*  = .585) (Table 2). The survival differential was also greater in studies conducted in Asia (OR_meta_ = 2.17, 95% CI = 1.68 to 2.81; HR_meta_ = 1.76, 95% CI = 1.03 to 3.01) (Table 2), followed by studies conducted in Europe (OR_meta_ = 2.02, 95% CI = 1.49 to 2.73; HR_meta_ = 1.46, 95% CI = 1.17 to 1.82) and in the United States (OR_meta_ = 1.58, 95% CI = 1.09 to 2.29; HR _meta_ = 1.52, 95% CI = 1.18 to 1.97) (Table 2). We observed statistically significant heterogeneity between studies included in these region-specific sensitivity analyses (Table 2).

We conducted sensitivity analyses restricted to cohort studies and those that used either multivariable adjustment or matching. The estimates in cohort studies were similar to the overall results (OR = 2.01, 95% CI = 1.56 to 2.58; HR = 1.45, 95% CI = 1.22 to 1.73). Estimates were also similar in studies using multivariable adjustment (OR = 1.99, 95% CI = 1.50 to 2.64; HR = 1.54, 95% CI = 1.29 to 1.84) or matching (OR = 2.12, 95% CI = 1.75 to 2.56) (Table 2).

Unadjusted odds ratio and hazard ratio estimates were overall greater than adjusted estimates (unadjusted OR_meta_ = 2.80, 95% CI = 2.23 to 3.52; unadjusted HR_meta_ = 1.84, 95% CI = 1.44 to 2.35) (Supplementary Figure 1, available online).

## Discussion

This meta-analysis of 129 840 patients across 42 studies demonstrates that hospitalized and unvaccinated COVID-19 patients with cancer have an increased risk of mortality compared with those without cancer (OR_meta_ = 1.93, 95% CI = 1.55 to 2.41; HR_meta_ = 1.54, 95% CI = 1.29 to 1.84). This is the largest meta-analysis to date, to our knowledge, assessing the risk of mortality in COVID-19 patients with cancer. Furthermore, this is the only meta-analysis that exclusively reports demographic and/or comorbidity-adjusted estimates for mortality, thus providing an appropriate methodological framework to assess cancer as an independent risk factor for mortality. We demonstrate with sensitivity analyses that when unadjusted estimates are used in the meta-analysis, the association between cancer and COVID-19 mortality is artificially higher than when adjusted estimates are used.

Patients with cancer may be at increased risk of COVID-19–related mortality because of intrinsic and treatment-related immune impairment. Lymphopenia has been reported as a clinical feature of COVID-19; thus, infection with the virus may exacerbate already dampened immune systems in these patients (62). Cancer patients recently treated with chemotherapy, targeted therapy, or immunotherapy have had statistically significantly higher COVID-19–related mortality rates compared with those not receiving recent treatment (63), demonstrating the role of cancer treatment in COVID-19 mortality. Our study also revealed a higher mortality differential among patients with hematologic malignancies. This patient population has numerous mechanisms for immune impairment. For example, patients with chronic lymphocytic leukemia are at risk of hypogammaglobinemia because of the disease and treatment with anti-CD20 agents (64). Defects in helper and cytotoxic T-cell function are also observed in these patients after treatment with fludarabine or alemtuzumab (64). Furthermore, patients receiving hematopoietic cell transplantation for hematologic malignancies require high-dose immunosuppression to prevent graft failure, which further impairs the immune response and delays immune recovery (65).

We acknowledge several limitations in this study, namely, that there was considerable heterogeneity between studies and evidence of publication bias. To address this, we conducted sensitivity analyses based on type of cancer, region, and study design, and we observed no statistically significant heterogeneity among the 6 studies focusing on hematologic malignancies. Among the remaining studies, the majority included mixed or unspecified cancers; thus, we were unable to further stratify results by solid tumor type. Regional subsets (Asia, Europe, United States) still demonstrated statistically significant heterogeneity. We suspect that a major source of heterogeneity in these studies may be cancer type or cancer stage. For example, patients with lung metastasis from any cancer type have been shown to have increased mortality and severe events from COVID-19 (66). However, a meta-analysis of 13 studies by Lei et al. (67) found that patients with lung cancer had similar COVID-19–related mortality than patients with other malignancies. There are limited data on mortality risk across other cancer types; thus, this is still an area of potential research. Other possible sources of heterogeneity include study design and choice of adjustment variables; however, our analysis of unadjusted estimates still demonstrated statistically significant heterogeneity. Other possible sources of heterogeneity we did not account for in our study include type of cancer treatment and time since last treatment, because of limited reporting of these variables. Factors such as facility, experience of medical staff, and patient immunity may play a role in the observed heterogeneity; however, the included studies do not provide such information. Additionally, the majority of included studies do not distinguish between patients who were admitted for a COVID-19 diagnosis vs those who tested positive during their hospital course. The latter subset is expected to have a less severe COVID-19 course. This may introduce heterogeneity that cannot be accounted for in the present study. There are also geographic differences in how both COVID-19 and cancer are diagnosed and managed, although we cannot obtain more information about these factors than what is available in the publications. Our sensitivity analyses based on continent suggest that the magnitude of association is different across continents, although these estimates are in the same range and similar to the overall estimate, thus confirming higher mortality in cancer vs noncancer patients with COVID-19. Finally, the included studies provide limited information on specific COVID-19 variants. The majority of included studies were conducted in 2020, during which the B.1.1.7 (Alpha), B.1.351 (Beta), and B.1.617.2 (Delta) variants first emerged between October and December (68). The Alpha variant was first detected in the United Kingdom in November 2020 and was the predominant strain there by December 2020, when it was first detected in the United States (68). The Beta variant was first discovered in South Africa during the country’s second COVID-19 wave in October 2020 before it spread to other regions and was associated with increased mortality than the prior wave (69). The Delta variant was not detected until December 2020 in India and is unlikely to be a major contributor to COVID-19 cases in the included studies. Such regional and temporal variations in predominant COVID-19 variants, which influence COVID-19 morbidity and mortality, were not accounted for in this study.

Our work addresses several methodological limitations in previous meta-analyses, such as the use of appropriate controls and the need for a laboratory-confirmed COVID-19 diagnosis. It also addresses limitations in statistical analysis, such as the need for adjusted estimates and the importance of conducting sensitivity analyses according to study design and geographic origin of each study. We also highlight several existing gaps and methodological shortcomings in the current literature on cancer and COVID-19: the lack of information on COVID-19 strain and COVID-19 treatment regimen, the need to adjust estimates for other risk factors for COVID-19 severity and mortality, and the need for information on each patient’s cancer history and treatment. There is also a need for standardized methods of patient selection, control selection, and data collection, as shown by the persistent heterogeneity observed in the meta-estimates, even when random effect methods are used for data aggregation.

We conclude that cancer is an independent risk factor for mortality in unvaccinated patients admitted for or diagnosed with COVID-19 during hospitalization. An important next step in future studies is to assess mortality among vaccinated COVID-19 patients with cancer to understand the role of vaccination in mitigating this risk. Such studies should stratify outcomes by type of vaccination, number of doses, and time since vaccination, in addition to adjusting for clinical and demographic characteristics known to be associated with COVID-19 mortality.

## Funding

National Institutes of Health/National Cancer Institute grant 5P30 CA196521-03 awarded to Ramon Parson, MD, PhD.

## Notes

**Role of the funder:** No role in the design of the study; the collection, analysis, and interpretation of the data; the writing of the manuscript; and the decision to submit the manuscript for publication.

**Disclosures:** The authors declare no potential conflicts of interest.

**Author contributions:** Conceptualization: ET; Data curation: MZ, NA, ET; Formal analysis: MZ; Supervision: ET; Writing—original draft: MZ, NA, ET; Writing—review and editing: MZ, ET.

## Supplementary Material

pkac063_Supplementary_Data

## Data Availability

No new data were generated or analysed in support of this research.
