# Supplementary material for: Methodological Considerations on COVID-19 Mortality in Cancer Patients: A Systematic Review and Meta-Analysis
Source: JNCI Cancer Spectr. 2022 Sep 1;6(5):pkac063. doi: 10.1093/jncics/pkac063 (PMC9619849; doi:10.1093/jncics/pkac063)
Supplement: pkac063_Supplementary_Data [file pkac063_supplementary_data.pdf]

SUPPLEMENTARY MATERIALS

Supplementary methods

The 2018 Mixed Methods Appraisal Tool (MMAT) was used by two researchers (NA, MZ) to perform a quality assessment of included studies (1). Each item was scored 0, 0.5 or 1 for studies that did not, somewhat, or fully met evaluation criteria respectively. An overall quality score was calculated for each study based on appropriateness of study design and collection methods, completeness of outcome data, risk of bias, and coherence. Scores ranged from 1 (low) to 5 (high) and no study was excluded on the basis of quality assessment (Supplementary table 1).

Additional meta-analyses were conducted using unadjusted OR and HR estimates if they were reported in the study or if sufficient data was provided to calculate them (Supplementary Figure 1 and 2). Publication bias was assessed using a funnel plot (Supplementary Figure 2).

References

1. Hong QN, Pluye P, Fàbregues S, Bartlett G, Boardman F, Cargo M, Dagenais P, Gagnon M-P, Griffiths F, Nicolau B, O'Cathain A, Rousseau M-C, Vedel I. Mixed Methods Appraisal Tool (MMAT), version 2018. Registration of Copyright (#1148552), Canadian Intellectual Property Office, Industry Canada.
2. Alamdari NM, Afaghi S, Rahimi FS, Tarki FE, Tavana S, Zali A, et al. Mortality Risk Factors among Hospitalized COVID-19 Patients in a Major Referral Center in Iran. Tohoku J Exp Med. 2020;252(1):73-84.
3. Alpert N, Rapp JL, Marcellino B, Lieberman-Cribbin W, Flores R, Taioli E. Clinical Course of Cancer Patients With COVID-19: A Retrospective Cohort Study. JNCI Cancer Spectrum. 2021;5(1):pkaa085.
4. Altuntas F, Ata N, Yigenoglu TN, Bascı S, Dal MS, Korkmaz S, et al. COVID-19 in hematopoietic cell transplant recipients. Bone Marrow Transplantation. 2021;56(4):952-5.
5. An C, Lim H, Kim D-W, Chang JH, Choi YJ, Kim SW. Machine learning prediction for mortality of patients diagnosed with COVID-19: a nationwide Korean cohort study. Scientific Reports. 2020;10(1):18716.
6. Anantharaman A, Dusendang JR, Schmittdiel JA, Harzstark AL. SARS-CoV-2 Clinical Outcomes in Patients with Cancer in a Large Integrated Health Care System in Northern California. Oncologist. 2021;26(3):e500-e4.
7. Başcı S, Ata N, Altuntaş F, Yiğenoğlu TN, Dal MS, Korkmaz S, et al. Outcome of COVID-19 in patients with chronic myeloid leukemia receiving tyrosine kinase inhibitors. J Oncol Pharm Pract. 2020;26(7):1676-82.

8. Bellan M, Patti G, Hayden E, Azzolina D, Pirisi M, Acquaviva A, et al. Fatality rate and predictors of mortality in an Italian cohort of hospitalized COVID-19 patients. Scientific Reports. 2020;10(1):20731.
9. Brar G, Pinheiro LC, Shusterman M, Swed B, Reshetnyak E, Soroka O, et al. COVID-19 Severity and Outcomes in Patients With Cancer: A Matched Cohort Study. Journal of Clinical Oncology. 2020;38(33):3914-24.
10. Ciceri F, Castagna A, Rovere-Querini P, De Cobelli F, Ruggeri A, Galli L, et al. Early predictors of clinical outcomes of COVID-19 outbreak in Milan, Italy. Clin Immunol. 2020;217:108509.
11. Cui N, Yan R, Qin C, Zhao J. Clinical Characteristics and Immune Responses of 137 Deceased Patients With COVID-19: A Retrospective Study. Front Cell Infect Microbiol. 2020;10:595333.
12. Docherty AB, Harrison EM, Green CA, Hardwick HE, Pius R, Norman L, et al. Features of 20 133 UK patients in hospital with covid-19 using the ISARIC WHO Clinical Characterisation Protocol: prospective observational cohort study. BMJ. 2020;369:m1985.
13. Eshrati B, Baradaran HR, Erfanpoor S, Mohazzab A, Moradi Y. Investigating the factors affecting the survival rate in patients with COVID-19: A retrospective cohort study. Med J Islam Repub Iran. 2020;34:88.
14. Esme M, Koca M, Dikmeer A, Balci C, Ata N, Dogu BB, et al. Older Adults With Coronavirus Disease 2019: A Nationwide Study in Turkey. J Gerontol A Biol Sci Med Sci. 2021;76(3):e68-e75.
15. Gude-Sampedro F, Fernández-Merino C, Ferreiro L, Lado-Baleato Ó, Espasandín-Domínguez J, Hervada X, et al. Development and validation of a prognostic model based on comorbidities to predict COVID-19 severity: a population-based study. Int J Epidemiol. 2021;50(1):64-74.
16. Guerra Veloz MF, Cordero Ruiz P, Ríos-Villegas MJ, Del Pino Bellido P, Bravo-Ferrer J, Galvés Cordero R, et al. Liver manifestations in COVID-19 and the influence of pre-existing liver disease in the course of the infection. Rev Esp Enferm Dig. 2021;113(2):103-9.
17. Gupta S, Hayek SS, Wang W, Chan L, Mathews KS, Melamed ML, et al. Factors Associated With Death in Critically Ill Patients With Coronavirus Disease 2019 in the US. JAMA Internal Medicine. 2020;180(11):1436-47.
18. Haase N, Plovsing R, Christensen S, Poulsen LM, Brøchner AC, Rasmussen BS, et al. Characteristics, interventions, and longer term outcomes of COVID-19 ICU patients in Denmark-A nationwide, observational study. Acta Anaesthesiol Scand. 2021;65(1):68-75.
19. Huang Y, Lyu X, Li D, Wang L, Wang Y, Zou W, et al. A cohort study of 676 patients indicates D-dimer is a critical risk factor for the mortality of COVID-19. PLoS One. 2020;15(11):e0242045.
20. Iftimie S, López-Azcona AF, Vicente-Miralles M, Descarrega-Reina R, Hernández-Aguilera A, Riu F, et al. Risk factors associated with mortality in hospitalized patients with SARS-CoV-2 infection. A prospective, longitudinal, unicenter study in Reus, Spain. PLoS One. 2020;15(9):e0234452.
21. Jiménez E, Fontán-Vela M, Valencia J, Fernandez-Jimenez I, Álvaro-Alonso EA, Izquierdo-García E, et al. Characteristics, complications and outcomes among 1549 patients hospitalised with COVID-19 in a secondary hospital in Madrid, Spain: a retrospective case series study. BMJ Open. 2020;10(11):e042398.
22. Joharatnam-Hogan N, Hochhauser D, Shiu KK, Rush H, Crolley V, Wilson W, et al. Outcomes of the 2019 novel coronavirus in patients with or without a history of cancer: a multi-centre North London experience. Ther Adv Med Oncol. 2020;12:1758835920956803.
23. Kim SW, Kim SM, Kim YK, Kim JY, Lee YM, Kim BO, et al. Clinical Characteristics and Outcomes of COVID-19 Cohort Patients in Daegu Metropolitan City Outbreak in 2020. J Korean Med Sci. 2021;36(1):e12.

24. Krause M, Douin DJ, Kim KK, Fernandez-Bustamante A, Bartels K. Characteristics and Outcomes of Mechanically Ventilated COVID-19 Patients-An Observational Cohort Study. J Intensive Care Med. 2021;36(3):271-6.
25. Kvåle R, Bønaa KH, Forster R, Gravningen K, Júlíusson PB, Myklebust T. Does a history of cardiovascular disease or cancer affect mortality after SARS-CoV-2 infection? Tidsskr Nor Laegeforen. 2021;140(2).
26. Lunski MJ, Burton J, Tawagi K, Maslov D, Simenson V, Barr D, et al. Multivariate mortality analyses in COVID-19: Comparing patients with cancer and patients without cancer in Louisiana. Cancer. 2021;127(2):266-74.
27. Martínez-López J, Mateos M-V, Encinas C, Sureda A, Hernández-Rivas JÁ, Lopez de la Guía A, et al. Multiple myeloma and SARS-CoV-2 infection: clinical characteristics and prognostic factors of inpatient mortality. Blood Cancer Journal. 2020;10(10):103.
28. Mehta V, Goel S, Kabarriti R, Cole D, Goldfinger M, Acuna-Villaorduna A, et al. Case Fatality Rate of Cancer Patients with COVID-19 in a New York Hospital System. Cancer Discovery. 2020;10(7):935-41.
29. Meng Y, Lu W, Guo E, Liu J, Yang B, Wu P, et al. Cancer history is an independent risk factor for mortality in hospitalized COVID-19 patients: a propensity score-matched analysis. Journal of Hematology & Oncology. 2020;13(1):75.
30. Mirani M, Favacchio G, Carrone F, Betella N, Biamonte E, Morenghi E, et al. Impact of Comorbidities and Glycemia at Admission and Dipeptidyl Peptidase 4 Inhibitors in Patients With Type 2 Diabetes With COVID-19: A Case Series From an Academic Hospital in Lombardy, Italy. Diabetes Care. 2020;43(12):3042-9.
31. Mohamed NE, Benn EKT, Astha V, Okhawere KE, Korn TG, Nkemdirim W, et al. Association between chronic kidney disease and COVID-19-related mortality in New York. World J Urol. 2021;39(8):2987-93.
32. Nogueira PJ, de Araújo Nobre M, Costa A, Ribeiro RM, Furtado C, Bacelar Nicolau L, et al. The Role of Health Preconditions on COVID-19 Deaths in Portugal: Evidence from Surveillance Data of the First 20293 Infection Cases. Journal of Clinical Medicine. 2020;9(8):2368.
33. Poterucha TJ, Elias P, Jain SS, Sayer G, Redfors B, Burkhoff D, et al. Admission Cardiac Diagnostic Testing with Electrocardiography and Troponin Measurement Prognosticates Increased 30-Day Mortality in COVID-19. J Am Heart Assoc. 2021;10(1):e018476.
34. Raines AM, Tock JL, McGrew SJ, Ennis CR, Derania J, Jardak CL, et al. Correlates of death among SARS-CoV-2 positive veterans: The contribution of lifetime tobacco use. Addict Behav. 2021;113:106692.
35. Ramachandran P, Kathirvelu B, Chakraborti A, Gajendran M, Zhahid U, Ghanta S, et al. COVID-19 in Cancer Patients From New York City: A Comparative Single Center Retrospective Analysis. Cancer Control. 2020;27(1):1073274820960457.
36. Sanchez-Pina JM, Rodríguez Rodriguez M, Castro Quismondo N, Gil Manso R, Colmenares R, Gil Alos D, et al. Clinical course and risk factors for mortality from COVID-19 in patients with haematological malignancies. Eur J Haematol. 2020;105(5):597-607.
37. Shah V, Ko Ko T, Zuckerman M, Vidler J, Sharif S, Mehra V, et al. Poor outcome and prolonged persistence of SARS-CoV-2 RNA in COVID-19 patients with haematological malignancies; King's College Hospital experience. Br J Haematol. 2020;190(5):e279-e82.
38. Shoumariyeh K, Biavasco F, Ihorst G, Rieg S, Nieters A, Kern WV, et al. Covid-19 in patients with hematological and solid cancers at a Comprehensive Cancer Center in Germany. Cancer Med. 2020;9(22):8412-22.
39. Sng CCT, Wong YNS, Wu A, Ottaviani D, Chopra N, Galazi M, et al. Cancer History and Systemic Anti-Cancer Therapy Independently Predict COVID-19 Mortality: A UK Tertiary Hospital Experience. Front Oncol. 2020;10:595804.

40. Stroppa EM, Toscani I, Citterio C, Anselmi E, Zaffignani E, Codeluppi M, et al. Coronavirus disease-2019 in cancer patients. A report of the first 25 cancer patients in a western country (Italy). Future Oncol. 2020;16(20):1425-32.
41. Thompson JV, Meghani NJ, Powell BM, Newell I, Craven R, Skilton G, et al. Patient characteristics and predictors of mortality in 470 adults admitted to a district general hospital in England with Covid-19. Epidemiol Infect. 2020;148:e285.
42. Yigenoglu TN, Ata N, Altuntas F, Bascı S, Dal MS, Korkmaz S, et al. The outcome of COVID-19 in patients with hematological malignancy. Journal of Medical Virology. 2021;93(2):1099-104.
43. Zandkarimi E, Moradi G, Mohsenpour B. The Prognostic Factors Affecting the Survival of Kurdistan Province COVID-19 Patients: A Cross-sectional Study From February to May 2020. Int J Health Policy Manag. 2022;11(4):453-8.

Supplementary Table 1. Quality review of studies included in the meta-analysis (1)

Study ID	There are clear research questions	The collected data allow to address the research question	Participants are representative of the target population	Measurements are appropriate regarding both the outcome and intervention (or exposure)	There are complete outcome data	Confounders are accounted for in the design and analysis	During the study period, the intervention is administered (or exposure occurred) as intended	Overall quality score (out of 5)
Alamdari et al. 2020 (2)	Yes	Yes	1	1	1	1	1	5
Alpert et al. 2021 (3)	Yes	Yes	1	1	1	1	1	5
Altuntas et al. 2020 (4)	Yes	Yes	1	1	0.5	0.5	1	4
An et al. 2020 (5)	Yes	Yes	1	1	1	1	1	5
Anantharaman et al. 2020 (6)	Yes	Yes	1	1	0.5	1	1	4.5
Başçı et al. 2020 (7)	Yes	Yes	0.5	1	0.5	1	1	4
Bellan et al. 2020 (8)	Yes	Yes	1	1	1	0.5	1	4.5
Brar et al. 2020 (9)	Yes	Yes	1	1	1	1	1	5
Ciceri et al. 2020 (10)	Yes	Yes	1	1	1	0.5	1	4.5
Cui et al. 2020 (11)	Yes	Yes	1	0.5	1	1	1	4.5
Docherty et al. 2020 (12)	Yes	Yes	1	0.5	1	1	1	4.5
Eshrati et al. 2020 (13)	Yes	Yes	1	0.5	1	1	1	4.5
Esme et al. 2020 (14)	Yes	Yes	0.5	0.5	0.5	1	1	3.5
Gude-Sampedro et al. 2020 (15)	Yes	Yes	1	0.5	0.5	1	1	4
GuerraVeloz et al. 2021 (16)	Yes	Yes	1	0.5	0.5	1	1	4
Gupta et al. 2020 (17)	Yes	Yes	1	1	1	1	1	5
Haase et al. 2021 (18)	Yes	Yes	1	1	1	0.5	1	4.5
Huang et al. 2020 (19)	Yes	Yes	1	0.5	1	1	1	4.5
Iftimie et al. 2020 (20)	Yes	Yes	1	0.5	0.5	1	1	4
Jimenez et al. 2020 (21)	Yes	Yes	1	0.5	1	1	1	4.5
Joharatnam-Hogan et al. 2020 (22)	Yes	Yes	1	0.5	0.5	1	1	4

Kim et al. 2021 (23)	Yes	Yes	1	0.5	1	1	1	4.5
Krause et al. 2020 (24)	Yes	Yes	1	0.5	1	0.5	1	4
Kvale et al. 2021 (25)	Yes	Yes	1	0.5	1	1	1	4.5
Lunski et al. 2021 (26)	Yes	Yes	1	0.5	0.5	1	1	4
Martínez-Lopez et al. 2020 (27)	Yes	Yes	1	0.5	0.5	1	1	4
Mehta et al. 2020 (28)	Yes	Yes	1	0.5	0.5	0.5	1	3.5
Meng et al. 2020 (29)	Yes	Yes	1	0.5	1	1	1	4.5
Mirani et al. 2020 (30)	Yes	Yes	1	0.5	1	0.5	1	4
Mohamed et al. 2021 (31)	Yes	Yes	1	0.5	1	1	1	4.5
Nogueira et al. 2020 (32)	Yes	Yes	1	0.5	0.5	1	1	4
Poterucha et al. 2021 (33)	Yes	Yes	1	1	1	1	1	5
Raines et al. 2021 (34)	Yes	Yes	1	0.5	0.5	1	1	4
Ramachandran et al. 2020 (35)	Yes	Yes	1	0.5	1	0.5	1	4
Sanchez-Pina et al. 2020 (36)	Yes	Yes	1	0.5	0.5	1	1	4
Shah et al. 2020 (37)	Yes	Yes	1	1	1	0.5	1	4.5
Shoumariyeh et al. 2020 (38)	Yes	Yes	1	1	1	1	1	5
Sng et al. 2020 (39)	Yes	Yes	1	1	1	1	1	5
Stroppa et al. 2020 (40)	Yes	Yes	1	0.5	0.5	1	1	4
Thompson et al. 2020 (41)	Yes	Yes	1	0.5	1	1	1	4.5
Yigenoglu et al. 2021 (42)	Yes	Yes	1	0.5	0.5	1	1	4
Zandkarimi et al. 2020 (43)	Yes	Yes	1	0.5	1	1	1	4.5

Supplementary Figure 1. Unadjusted risk of COVID-19 mortality in cancer vs. noncancer patients in studies reporting odds ratios (A) or hazard ratios (B).

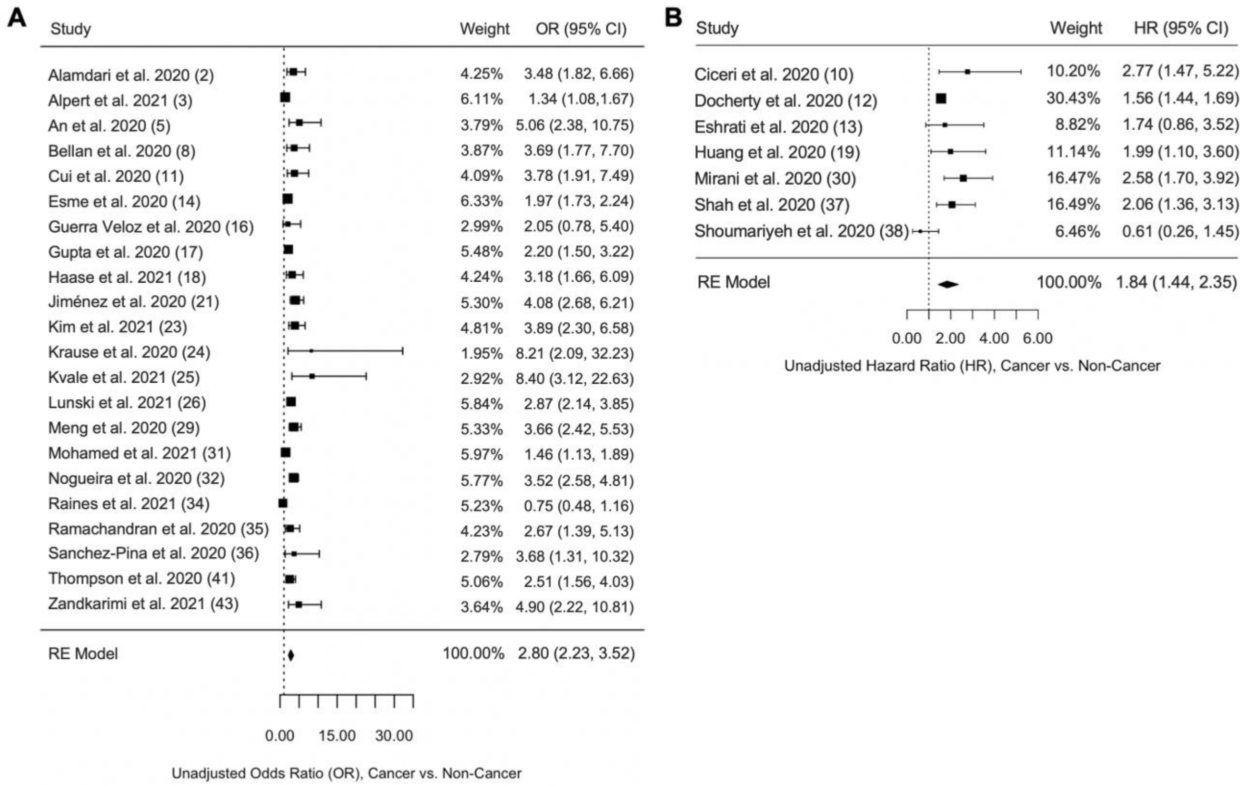

Forest plot for **A)** studies reporting odds ratio (22 studies) and **B)** studies reporting hazard ratio (7 studies). OR = odds ratio. HR = hazard ratio. CI = confidence interval. RE = random effects. The reference group is the non-cancer group.

Supplementary Figure 2. Funnel plot to assess for publication bias in studies reporting odds ratios (A) or hazard ratios (B) for COVID-19 mortality in cancer vs. noncancer patients.

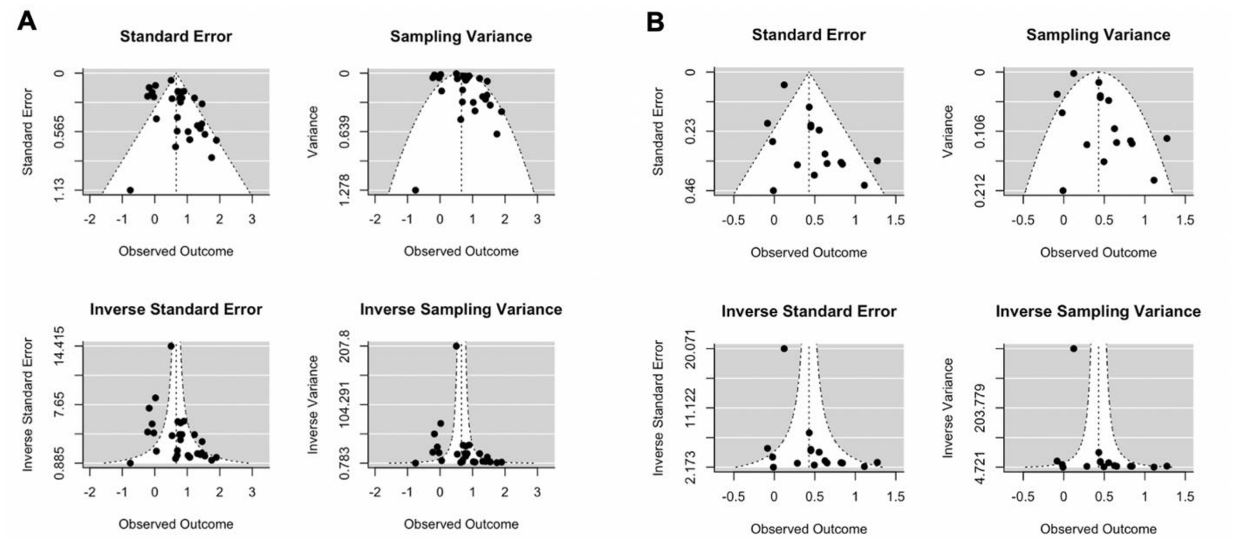

Funnel plot for **A)** studies reporting odds ratio (28 studies) and **B)** studies reporting hazard ratio (16 studies).
